# Molecular Changes on Maternal–Fetal Interface in Placental Abruption—A Systematic Review

**DOI:** 10.3390/ijms22126612

**Published:** 2021-06-21

**Authors:** Monika Bączkowska, Magdalena Zgliczyńska, Jan Faryna, Ewa Przytuła, Błażej Nowakowski, Michał Ciebiera

**Affiliations:** 1 Center of Postgraduate Medical Education, Second Department of Obstetrics and Gynecology, 01-809 Warsaw, Poland; monika.baczkowski@gmail.com (M.B.); zgliczynska.magda@gmail.com (M.Z.); 2 Department of Pathology, Bielański Hospital, 01-809 Warsaw, Poland; janfaryna@poczta.fm (J.F.); ewa.przytula.szpital@gmail.com (E.P.); 3 The Greater Poland Cancer Center, Department of Surgical, Oncological, and Endoscopic Gynecology, 61-866 Poznań, Poland; blazej@me.com

**Keywords:** placental abruption, decidua, endometrium, myometrium, placenta, maternal–fetal interface, immunology

## Abstract

Placental abruption is the separation of the placenta from the lining of the uterus before childbirth. It is an infrequent perinatal complication with serious after-effects and a marked risk of maternal and fetal mortality. Despite the fact that numerous placental abruption risk factors are known, the pathophysiology of this issue is multifactorial and not entirely clear. The aim of this review was to examine the current state of knowledge concerning the molecular changes on the maternal–fetal interface occurring in placental abruption. Only original research articles describing studies published in English until the 15 March 2021 were considered eligible. Reviews, book chapters, case studies, conference papers and opinions were excluded. The systematic literature search of PubMed/MEDLINE and Scopus databases identified 708 articles, 22 of which were analyzed. The available evidence indicates that the disruption of the immunological processes on the maternal–fetal interface plays a crucial role in the pathophysiology of placental abruption. The features of chronic non-infectious inflammation and augmented immunological cytotoxic response were found to be present in placental abruption samples in the reviewed studies. Various molecules participate in this process, with only a few being examined. More advanced research is needed to fully explain this complicated process.

## 1. Introduction

### 1.1. Placental Abruption—Overview and Epidemiology

Placental abruption is defined as the early complete or partial separation of the placenta from the lining of the uterus with the development of a retroplacental hematoma before delivery [1]. Placental abruption is a relatively infrequent perinatal complication. However, it is associated with a marked risk of maternal and fetal mortality and requires emergent management [2]. The complication is characterized by a sudden onset and frequently occurs when a patient is not in the hospital. An analysis by Ananth et al. (2015) showed that placental abruption rates tended to decline in the majority of developed countries [3] where the incidence ranged from 0.4 to 1% [1]. As regards to developing countries, the reported risk was higher [4], especially in the case of multiparas, or patients with a history of cesarean section, previous abortion, or placental abruption [5]. Placental abruption patients are more commonly referred to tertiary centers, but actually, each obstetrician may face such a complication in gestations which were initially physiological. This is due to the fact that placental abruption may also occur in patients in labor (about 0.3% of all term deliveries) [6].

Placental abruption may be associated with a variety of complications, even in the case of an instant availability of highly qualified medical personnel and efficiency in saving the mother and the child. The complications are related to iatrogenic prematurity, poor condition of the newborn at birth necessitating hospitalization in the neonatal intensive care unit, prolonged hospital stay and an intervention in the mother. The examples of more common complications of placental abruption include severe hemorrhage, fetal demise, maternal death, premature labor, low birth weight, repetitive transfusions, coagulopathy, or invasive procedures including hysterectomy [1]. According to a systematic review by Downes et al. (2017) placental abruption might also be associated with the elevated risk of sepsis, amniotic fluid embolism, acute kidney injury, severe respiratory distress, encephalopathy, and maternal intensive care unit admission [7]. It also affects a long term prognosis—placental abruption is a significant risk factor for long term maternal cardiovascular disease mortality in a follow-up period of over a decade [8,9].

The exact cause of placental abruption is not entirely clear, and several risk factors are known. Overall, the risk factors of developing placental abruption include advanced maternal age, a history of placental abruption, the use of stimulants, increased arterial pressure (with its sequelae, such as preeclampsia (PE), or fetal growth restriction (FGR)), infectious agents, polyhydramnios, preterm or prolonged rupture of membranes, a history of cesarean section, multiple pregnancies or thrombophilia [1,10]. As regards the cases of placental abruption at term, it was demonstrated that they were significantly associated with pregnancy-induced hypertension, non-vertex presentation, FGR, polyhydramnios and advanced maternal age [6]. Placental abruption may also occur in the case of an abdominal injury in a pregnant woman, e.g., during a vehicle accident or a fall. Some authors also reported the possible influence of stress [11], or heavy physical exertion [12]. Furthermore, several genetic loci were identified that might play a part in placental abruption risk change.

For example, Qiu et al. found that rs2899663 single nucleotide polymorphism of the *RORA* gene was associated with a 21% reduction in the odds of placental abruption. Most of the data in this area have been summarized recently. According to a well performed genome-wide association study and a meta-analysis of genome-wide association studies by Workalemahu et al. from 2018, the genetic loci suggestively associated with placental abruption included rs4148646 and rs2074311 in *ABCC8*, rs7249210, rs7250184, rs7249100 and rs10401828 in *ZNF28*, rs11133659 in *CTNND2*, and rs2074314 and rs35271178 near *KCNJ11*, rs76258369 near *IRX1*, and rs7094759 and rs12264492 in *ADAM12*. Additional analyses performed by the authors revealed that the majority those genes are connected to trophoblast-like cell interaction, or in the endocrine or cardiovascular systems or cellular function pathways. However, their role still needs further research [13].

### 1.2. Placental Abruption—Pathophysiology

The etiopathogenesis of placental abruption is multifactorial. According to the current concept of ischemic placental disease, placental abruption is one of the manifestations of impaired placental function comprising PE and FGR. According to the available literature, the pathophysiological mechanisms leading to the occurrence of such complications include uteroplacental ischemia (the placenta becoming underperfused) and placental insufficiency beginning during placental implantation [14,15]. It is believed that those various conditions represent the same disease processes with advancing gestation [14,15].

In the majority of cases, placental diseases are related to the abnormalities of the vascular and immune system. The disruptions lead to necrosis, inflammation, vascular problems and, ultimately, to placental abruption [16,17]. Physiological changes in the immune tolerance process are observed during each stage of pregnancy and labor [17]. The first reports considering labor as a non-infectious inflammatory process were published a long time ago, but the issue is still valid and insufficiently studied due to its multifactorial character. It is currently known that it is the temporary activation of the maternal immune system in which unique suppression is maintained throughout pregnancy [18,19].

Immune tolerance is necessary at the interface between the mother and fetus [20]. The maternal–fetal interface includes the extravillous trophoblast, the decidual stromal cells that house the maternal immune cells (i.e., T cells, uterine natural killer cells, macrophages, dendritic cells) and the uterine vessels with their endothelium. Each of the components may individually participate in the pathogenesis of preterm birth [21]. The immune system cells and functional regulation are involved in controlling the normally silent inflammatory process of the human uterine decidua, which is a state of very delicate balance [18,22]. Immune cells, such as decidual natural killer (dNK) cells, macrophages, B cells, T cells, dendritic cells, and natural killer T cells (NKTs), are the key players in this process [22]. It is known that chronic placental inflammation might occur in even 10% of pregnancies. However, this inflammation is generally not associated with a documented infection [23]. Numerous authors claimed that placental abruption was associated with a chronic non-infectious inflammatory process in which placental abruption was an easily observable end point, and not an acute phenomenon occurring in isolation [14]. Inflammatory disorders affecting the placenta represent a diverse category of pathological processes leading to adverse perinatal outcomes. For example, maternal and fetal inflammatory responses are related to the clinical diagnosis of chorioamnionitis (CA), an acute type of inflammation that might be associated with potential respiratory and neurological diseases. Conversely, the following chronic placental inflammations may occur: chronic villitis of unknown etiology, chronic deciduitis, chorionitis or intervillositis [24].

The immunological alterations occurring in placental abruption remain mostly unknown, yet some of them have already been established. The disruption of the NKs and T cell balance may result in preterm placental abruption [16,17]. According to the available data, an increased risk of placental abruption was observed to be accompanied by an increased infiltration of macrophages and neutrophilic cells in the uterus [25,26,27,28,29,30]. A high proportion of experts claimed that placental abruption was particularly related to the accumulation of cytotoxic response resulting from the insufficient immunosuppressive activity of the decidua [30,31].

Recent studies also suggested an important role of the cytosolic multiprotein oligomers responsible for the activation of inflammatory responses. Therefore, they were called inflammasomes in the pathophysiology of placental abruption. Many different factors are connected with the fine-tuned regulation of inflammasome assembly and the final effect [32] in this area. For example, NOD-like receptor proteins (NLRPs), e.g., NLRP7, are important in reproductive losses [33]. According to Abi Nahed et al. (2019), NLRP7 was expressed by trophoblast cells and regulated by hypoxia. NLRP7 was found to play a key role in trophoblastic cell proliferation, migration, and invasion. NLRP7 deregulation was suggested to be connected to the occurrence of severe pregnancy outcomes which might precede placental abruption.

It is worth mentioning the abnormal separation of the placental tissue, which may partially be included in placenta accreta spectrum, i.e., a range of the pathologic adherence of the placenta, including placenta increta, placenta percreta, and placenta accreta [34,35]. The spectrum and the described situations are currently referred to as the concept of the biologically defective decidua rather than the primarily abnormally invasive trophoblast [35]. Conversely, it is worth presenting the condition opposite to placental abruption—the retained placental tissue (RPT) in which, for an unclear reason, some placental tissue is not completely separated from the uterine wall. RPT is a condition in which all or a part of the placenta or membranes failed to separate from the uterine lining and were retained within the uterus [36]. It also includes the abovementioned placenta accreta spectrum [37]. A high proportion of those situations are probably associated with the insufficient accumulation of cytotoxic response factors resulting from the immunosuppressive hyperactivity of the decidua [17,29]. Therefore, a comparison between the immunological picture of the decidua in the case of placental abruption and RPT seems to be an interesting issue in available and future research.

A successful pregnancy involves various interactions between trophoblasts and maternal immune cells. These interactions allow the development of a new life in the uterus while the mother’s immune system remains intact [31]. The planned systematic review aims at the determination of the molecular basis of the clinical diagnosis of placental abruption and identifying pathways and factors leading to this complication. The obtained data will facilitate a better understanding of the phenomenon and the improvement of diagnostic and therapeutic opportunities for patients at risk of developing placental abruption.

## 2. Material and Methods

The methods of the analysis and inclusion criteria were specified in advance. The inclusion and exclusion criteria are summarized in Table 1.

The above criteria were strictly balanced in order to minimize the overall bias of this review. Animal studies were excluded to ensure the examined processes indeed occurred in humans. Studies concerning in vitro fertilization were excluded to minimize the influence of iatrogenic interventions on the maternal-fetal unit. To establish the data concerning the molecular changes on the maternal–fetal interface, studies examining only blood serum, genetic mutations or histopathological examination only were excluded.

Two electronic databases were used in this systematic search: MEDLINE (through PubMed) and Scopus. The search strategy for each database is presented in Table S1. The last search was performed on the 15 March 2021 on each database. The authors also reviewed the reference lists of selected studies; however, no additional records meeting the inclusion criteria were noted. It was not necessary to contact the authors of retrieved research articles for additional information.

A systematic literature search retrieved 708 citations. The Scopus search was indexed to exclude the MEDLINE articles. The remaining duplicates were removed using the automatic EndNote X9 (Clarivate Analytics, Philadelphia, PA, USA) duplicate finder, followed by a manual search. An eligibility assessment was performed independently in an unblinded standardized manner by two reviewers. Details regarding the selection process are summarized in a custom-built PRISMA flow chart in Figure S1. In the next step, the authors collected data from each selected study using a self-developed data extraction sheet. One of the authors extracted the following data from the included studies (M.B.) and the second author checked the extracted data (M.Z.) Information was extracted from each included trial on the characteristics of the material examined (the source and amounts of tissue samples, characteristics of cell cultures or models); the list of investigated proteins; and the outcome and effects observed in placental abruption samples. If the research was conducted in cell cultures or models of the physiological process and there were no samples from patients with placental abruption, the field “Effect observed in placental abruption samples” was marked by the note “POSSIBLE EFFECT”. Any disagreements were resolved through discussion and consensus; if no agreement could be reached, the third and sixth author decided (M.C. and J.F.).

The risk of bias for the interpretation of the data was analyzed with the use of the modified Office of Health Assessment and Translation (OHAT) Risk of Bias Rating Tool for Human and Animal Studies (https://ntp.niehs.nih.gov/ntp/ohat/pubs/riskofbiastool_508.pdf, accessed on the 11 April 2021). The OHAT Tool was developed to enable risk of bias assessment in all kind of studies. Six types of bias (selection, confounding, performance, attrition/exclusion, detection, and selective reporting) are rated based on the four-point scale: “definitely low risk of bias”, “probably low risk of bias”, “probably high risk of bias” and “definitely high risk of bias”. Other possible sources of bias, not included in the scale, have been discussed in a separate paragraph.

## 3. Results

As a result of the described procedure, 22 articles that met all the inclusion criteria were retrieved [20,21,27,29,38,39,40,41,42,43,44,45,46,47,48,49,50,51,52,53,54,55]. Extracted data included in this systematic review are collected in Table S2.

All of the articles finally selected for the review were original studies published in English. All the studies were conducted in vitro. In 11 articles, the effect in placental abruption was marked as “possible”, as the research was conducted in cell culture or a model and there were no samples from patients with placental abruption. In several studies [21,27,42,49,50,51,52,54,55], the researchers treated the samples (endometrial cells, placental samples, decidual cells or decidual cell cultures, fetal membranes or fetal membrane models, amnion cell cultures, human endothelial endometrial cell cultures, myometrial samples or myometrial cell samples) with THR and studied the molecular effects of this intervention. We have included these studies, as they may be considered the models of a situation actually occurring in the case of placental abruption.

The included studies involved samples from 581 participants, blood samples from 134 participants and 10 cell cultures/models. In one study, the number of participant samples was not described. The median number of tissue samples included in each study (apart from blood samples and cell cultures) was 34, and the average was 38. The main inclusion criteria entailed studies describing immunological changes occurring in the maternal–fetal interface in placental abruption, although we excluded studies in which only blood serum tests were conducted (studies where blood sample tests were a part of the research were not excluded). Summarized outcomes of all the included studies are available in Supplementary Materials in Table S2.

The first studies concerning the problem were conducted over 30 years ago by Altshuler et al. (1983) [38] concerning the elevated human placental lactogen levels in the placentas from placental abruption patients and by Ching et al. (1987) [39] concerning the changes of opiate receptors in opiate users—a high placental abruption risk group. However, the available data are still scarce and even with a variety of studies, many questions still remain unanswered.

### 3.1. Molecular Effects of Thrombin in Placental Abruption

Bleeding is clinically inseparably associated with placental abruption. Apparently, it may be regarded both as the cause and as the result of placental abruption. Even first trimester bleeding, according to different studies, may increase the risk of placental abruption itself [56,57,58], as well as preterm birth (PTB) risk, 2-fold [59] and the preterm premature rupture of membranes (PPROM) risk, 8-fold [60]. Even in the instance of a lack of clinically symptomatic bleeding, decidual hemorrhage in histopathological samples was found eight times more often in PTB than in term birth [61]. Bleeding is a source of blood clotting factors, immune cells and mediators, which start cascades of events at the molecular level, clinically leading to placental abruption, PPROM or PTB.

During placental abruption, the decidua is exposed to bleeding-derived clotting factors, thrombin (THR, F2) being one of the most abundant [27,54,55] and best examined. THR is a serine proteinase [62] and the main effector protease of coagulation [21]. It mostly acts via protease-activated receptors 1 (PARs)–high-affinity, transmembrane THR receptors [62,63]. THR activates PAR1 (F2R), PAR3 (F2RL2), and PAR4 (F2RL3), but not PAR2 (F2RL1) [62]. PAR1 seems to be the most important of the PARs family and was expressed by all parts of the maternal–fetal interface despite amnion cells [51,54,64,65], with its expression higher in pregnancy compared to non-pregnancy, and highest during delivery [54,66].

The dysregulation and increase of THR activity has the potential to cause PTB [54]. It also plays a pivotal role in abruption-associated PPROM [50]. Thrombin-antithrombin complex levels were even identified as the predictors of the PTB and/or PPROM with high sensitivity and specificity [67,68,69]. THR exerts its effect through a variety of mechanisms.

#### 3.1.1. Thrombin-Induced Myometrial Contractions

It is known from the clinical practice that bleeding—manifested or latent—causes uterine contractions. It may be understood as a mechanism protecting the mother and expelling the fetus in case of the danger of a pathological intrauterine bleeding. Nishimura et al. (2020) [54] focused on the aspect of THR which effects myometrial cells. They proved that myometrial contractions were induced by THR via two mechanisms: through the direct activation of myosin II [54,70] and through an indirect increase in prostaglandin synthesis [54]. The first mechanism is the effect of THR signaling via PAR1, which activates myosin light chain kinase (MLCK, MLYK) and Rh—associated protein myosin light chain kinase (ROCK)—which leads to the regulatory light chain of myosin II (MLC2, MYL9) phosphorylation, myosin II activation and contraction. Interestingly, the contraction was not the result of the inhibition of myosin phosphatase [54]. The observed increase in prostaglandin and interleukin synthesis involved prostaglandin E2 (PGE2), prostaglandin F2α (PGF2α) and interleukin-1β (IL-1β) with a concomitant decrease in prostaglandin receptors: the prostaglandin F2α receptor (PTGFR) and the prostaglandin E2 receptor 3 (PTGER3) expression. They also observed an increase in the COX2 (PTGS2) expression, and no effect on the oxytocin receptor (OXTR), gap junction alpha-1 protein (GJA1 or CX-43 or connexin 43) or prostaglandin E2 receptor 1 (PTGER1). Interestingly, they also observed a significant decrease in the expression of progesterone receptor type A (PR-A) and progesterone receptor type B (PR-B), as well as total progesterone receptor (total PR), apparently responsible for the functional progesterone withdrawal and responsible for both initiation and further strengthening of contractions. Their findings seemed consistent with the clinical observations and THR functions in physiological labor, where an increase in maternal circulating THR is observed in the third stage of labor and is responsible for the contraction of the myometrium [54].

#### 3.1.2. Thrombin-Induced Fetal Membrane Weakening

THR induces fetal membrane (FM) weakening in several different ways. Following Kumar et al. (2011) [49], THR is capable of the direct weakening of the isolated amnion. It was consistent with the findings of Rosen et al. (2002) [42] concerning the THR’s direct enhancement of matrix metalloproteinase 1 (MMP1), and Puthiyachirakkal et al. (2013) [51] concerning the direct weakening effect of THR via PAR1. According to Kumar et al. (2011) [49] and Lockwood et al. (2012) [27], THR also exerted an indirect weakening effect on FM via choriodecidua-derived cytokines (among others IL-8 [27,42,71,72]), unidentified soluble activators [51] or chemoattracted neutrophils. According to Sinkey et al. (2020) [55] and Kumar et al. (2014) [52] the granulocyte-macrophage colony-stimulating factor (GM-CSF, CSF2) is one of the most important mediators of this process. Interestingly, GM-CSF, a known inducer of those proteases [73,74,75], was detected only on the maternal side of FM [52] and transduced the signal from both THR and TNFα [55]. Decidual GM-CSF acted through a trophoblast-expressed granulocyte-macrophage colony-stimulating factor receptor (colony-stimulating factor 2 receptor or CSF2R) [55]. Decidual signaling pathways involved in THR-induced CSF-2 expression are mitogen-activated protein kinase: a subfamily of extracellular signal-regulated kinases (ERK 1/2 MAPK), nuclear factor kappa-light-chain-enhancers of activated B cells (NF-κB), not mitogen-activated protein kinase: subfamily of p38 mitogen-activated protein kinases (p38 MAPK) or mitogen-activated protein kinase: subfamily of c-Jun N-terminal kinase (JNK MAPK) [50,55]. As regards trophoblasts, CSF-2 activates signal transducers and is an activator of transcription 5 (STAT5), inhibits p38 MAPK, and exerts no effect in the signal transducer and activator of transcription 3 (STAT3), ERK1/2 MAPK, activator protein 1 (Akt or PKB or protein kinase B or serine/threonine-specific protein kinase or AP-1), and NF-κB p65 (one of NF-κB components) [55]. Only high concentrations of CSF-2 caused an increase in IL-1β and MMP9, but despite the concentrations there was no impact on MMP3, MMP7, IL-6, IL-8. Sinkey et al. (2020) [55] also made an interesting hypothesis. They claimed that it might be the exosomes that mediated paracrine CSF2-CSF2R interactions between decidual cells and trophoblasts at the maternal–fetal interface. All those mechanisms led to MMP induction [42,51,72,76,77,78,79], explained by a few different hypotheses. MMPs can be derived from chemoattracted neutrophils. Uszynski et al. (2004) [44] described the influence of THR on the urokinase plasminogen activator/urokinase plasminogen activator receptor/plasmine (uPA/uPAR/plasmine) proteolytic system, resulting in MMPs enhancement. The results are inconsistent with the findings of Norwitz et al. (2007) [45], who proved the irrelevance of the mechanism, finding the direct action of THR via PAR-1 more important [72,80]. Lockwood et al. (2012) [50] also estimated that THR caused a decrease in progesterone receptors, and a decrease in PR-binding activity. This down-regulation was proven to occur via ERK 1/2 MAPK phosphorylation. Finally, it led to a functional progesterone withdrawal, suspected to be an important initiator of abruption-associated PTB. The overall THR effects on MMPs expression are presented in Table 2. The molecules inhibiting this fetal membrane weakening effects were found to be medroxyprogesterone acetate (MPA) [55], progesterone (P4), or 17-αhydroxyprogesterone (17α-OHP) [81], dexamethasone or pure progestin [55], NF-kB inhibitor, and metal chelating agent, lipoic acid [49]. These protective properties need further clinical investigation, but they are a potential target in the treatment.

### 3.2. Inflammation-Derived Pathways in Placental Abruption

A link between inflammation and bleeding was reported by many researchers [21,40,47,48,53]. Moreover, according to Mhatre et al. (2016) [21], a similar inflammatory response may be driven both by lipopolysaccharide (LPS) and THR, achieving a multiplied effect when acting together (see Table 3). THR may also be produced from the fetal membrane, not only from intrauterine bleeding, but also due to a bacterial infection [65,82]. Such a correlation creates a dangerous vicious circle, inevitably heading to pregnancy termination. Any therapeutic “brakes” on this process may be beneficial for the preservation of pregnancy.

An inflammation or infection was clinically confirmed to be associated with an increased risk of placental abruption [83]. In fact, the features of sterile inflammatory changes were found in many cases of placental abruption [27]. The mechanisms and pathways by which inflammation found in the reproductive tract was expressed, included the promotion of leukocyte trafficking and the disruption of vascular integrity, leading to proinflammatory molecule production [47]. The fact that inflammatory changes are suspected to occur already in the first weeks of pregnancy or even before conception and remain latent for weeks or months [84] makes the issue even more complex. According to Snegovskikh et al. (2009) [47], one of key mediators is a vascular endothelial growth factor (VEGF) with its receptors (fms-like tyrosine kinase-1 (FLT1), kinase insert domain receptor 2 (KDR2 or VEGFR-2 or vascular endothelial growth factor receptor 2)) and co-receptors (neuropilin-1, -2). Both VEGF and neuropilins (but not Flt-1 and KDR2) were upregulated after stimulation with IL-1β, which provided an explanation for how an intra-amniotic infection might increase the risk of placental abruption.

The existence of tissue-specific angiogenic factors has been postulated for many years [6,7,8,9]. However, it has been recently confirmed that such a factor, named endocrine gland-derived vascular endothelial growth factor/prokineticin 1 (EG-VEGF/PROK1), had been finally characterized.

In the last decade, the endocrine-gland-derived vascular endothelial growth factor (EG-VEGF), also called prokineticin 1 (PROK1), emerged as a specific placental angiogenic factor that controls numerous aspects of normal and pathological placental angiogenesis, such as recurrent pregnancy loss (RPL), gestational trophoblastic diseases (GTD), FGR and PE.

EG-VEGF factor and its receptors PROKR1 and PROKR2 are among recently investigated angiogenic factors that are specific to the placenta. Authors should refer to the literature concerning EG-VEGF in the placenta and in relation to pregnancy pathologies that lead to FGR and preeclampsia, two key pathologies that are associated with placental abruption. Compared to VEGF, EG-VEGF and its receptors are less ubiquitous, which may open huge perspectives in their consideration in the etiology of placental abruption [85].

During early pregnancy, EG-VEGF/PROKR1 peaks at 8–11 weeks of gestation and then gradually decreases by the end of the first trimester, whereas PROKR2 expression is maintained over the first trimester. EG-VEGF promotes the proliferation of anchoring trophoblasts and inhibits early EVT migration and invasion. In the first trimester human placenta, anchoring trophoblastic plugs obstruct the spiral arteries and prevent maternal oxygenated blood from entering into the intervillous space [85,86].

PK1 may have a novel role as a mediator of the inflammatory response in the placenta. PK1 was found to induce a time-dependent increase in the expression of IL-8 [87] and COX-2.

The role of cyclooxygenases (COXs) at the time of implantation and during pregnancy is well known, with successful implantation being associated with the prostaglandin (PGs) induction by COX1 and COX2 [88,89]. A precise balance between COX-negative and COX-positive cells is necessary to ensure the adequate production of PGs and to allow a physiological evolution of pregnancy [48]. Placental COX1 and COX2 activities were reported to be decreased in case of PE [90,91], while fetal COX2 polymorphism was suspected to contribute to the development of FGR [92]. The role of COXs in placental abruption remains unclear and the research is still ongoing. Following Avagliano et al. (2011) [48], the imbalance of COXs seems to be unassociated with the pathogenesis of placental abruption, while Singh et al. (2019) found an increased expression of COX2 in the amnion in placental abruption [53]. Furthermore, Nishimura et al. (2020) found an increased expression of COX2 in the myometrium treated with THR [54]. This subject needs further research.

Moreover, nitric oxide, peroxynitrite and the enhanced expression of NO synthase (NOS), which are footprints of an inflammation and are found in the placenta of patients with CA, also seem to be present in samples collected from placental abruption patients. Probably the cascade of placental injury is similar in cases of CA and placental abruption. According to Nakatsuka et al. (1999), nitric oxide and its metabolites may play an important role in this cascade [40]. Activated macrophages and polymorphonuclear leukocytes are their possible sources [93]. They are present in both clinical situations. Interestingly, only an increase in inducible NOS (iNOS), but not in endothelial NOS (eNOS), was observed in the malfunctioning placentas of patients with placental abruption or CA.

As presented in Table S4, the increased infiltration of different immune cells and the expression of prostaglandins, chemokines, receptors and co-receptors are present at the maternal–fetal interface in placental abruption.

### 3.3. Decidual Immunoreactivity

Decidua plays a crucial role in the process of human reproduction. The proper placentation process depends on the adequate invasion of the extravillous trophoblast into the decidualized endometrium and the modification of spiral arteries. The disruptions of this process may lead to all of the main causes of perinatal or maternal morbidity and mortality including FGR, PE, PTB and placental abruption. Decidualization causes the change in human endometrial stromal cell expression of tissue factor (TF), type-1 plasminogen activator inhibitor (PAI-1), matrix metalloproteinases (MMPs) and others [94], which is one of the mechanisms ensuring hemostasis. The decidua has also a unique property of the physiological coexistence of activated immune cells with the decidual stromal cells. It regulates immune cell infiltration and their activity [17]. These cellular interactions are responsible for the immune tolerance of pregnancy and are crucial for its survival. They extend beyond the implantation up to the labor and determine placental detachment. The placental abruption, as well as the parturition, is associated with insufficiency of decidual suppressive activity and the accumulation of cytotoxic immune cells in the decidua. The responsibility for this insufficiency was assigned to reduced levels of immunomodulating membrane proteins [20]. Placental abruption seems to occur when molecular changes at the maternal–fetal interface are terminated precociously before uterine cervical ripening and a child’s expulsion takes place.

Receptor-binding cancer antigen expressed on SiSo cells (RCAS1, EBAG9) is a type II membrane protein described in the context of placental abruption [20,29]. It is expressed in the extravillous cytotrophoblast, villus histiocytes and uterine endometrium, as well as in various human cancer cells [95,96,97,98,99]. It is a ligand for a putative lymphocyte receptor [95,96,97,99]. It plays an important role in the maintenance of maternal immune tolerance and the pregnancy itself [20,98], induces apoptotic cell death [95,100] and supports avoiding immune recognition and escaping from immune surveillance [20]. It suppresses the decidual natural killer cells (dNK), natural killer cells (NK), and cytotoxic lymphocytes (CTLs) and inhibits active cytotoxic cells [98], finally resulting in the inhibition of the maternal immune attack on the fetal antigen [101]. According to the studies on its expression in placental abruption, it was shown that the expression of RCAS1 was lower in placental abruption in comparison with elective cesarean section (ECC) or cases of remained placental tissue (RPT), and it was similar to vaginal delivery (VD) or cases of maternal rejection [20,29,98,102,103,104]. No statistically significant differences were recognized during labor progress [29]. Such an insufficiency was accompanied with the excessive accumulation and activity of CD3+ and CD56+ cells in the decidua [29]. The interesting concept of this research was to compare the placental abruption samples to RPT samples. Such pathologic situations are the opposite deviation of the same continuum process of the placenta separation, with the 3rd stage of labor being the physiological balance between both. Therefore, it confirmed the hypothesis of the suppressive and anti-inflammatory role of the RCAS1 in the decidua.

Metallothionein (MT) is a low molecular weight protein with a high cysteine content and a marked affinity for divalent metals [105,106,107]. It inhibits cell apoptosis [46] via the change of cell sensitivity to induced apoptosis [105,108,109,110,111]. It decreases caspase-3 activation and the mitochondrial-originating cytochrome c level [112]. It is a crucial mediator of the coexistence of the immune and decidual cells [17,46,113]. MT is suspected to participate in the maintenance of homeostasis in the reproductive tissues [46]. The levels of MT immunoreactivity are regulated by numerous molecules (hormones; cytokines: interleukin 1 (IL-1) [112], interleukin 6 (IL-6) [114], tumor necrosis factor α (TNF-α) [115], interferon gamma (INF-γ) [116] and stress-induced factors, i.e., heavy metals [117,118]). Galazka et al. (2008) [46] studied the role of MT in placental abruption. The expression of MT was higher in placental abruption, and in eutopic and ectopic decidua with advanced labor [46] or in spontaneous abortion [119], with no significant differences with respect to the progression of labor [46]. It confirmed the hypothesis of MT being a homeostasis mediator, increasing in the presence of enhanced cytotoxicity occurring in placental abruption.

### 3.4. Links between Placental Abruption and Preterm Premature Rupture of Membranes

A strong clinical association was observed between placental abruption and subsequent PPROM [42]. It was found that PPROMs commonly accompanied abruptions [55]. Between 22 and 32 weeks of gestation, abruption occurred in over one-third of PPROM cases [9,61,120]. Occult decidual hemorrhage was reported in 72 of 192 (37.5%) patients with PPROM compared to only 1 of 108 (0.8%) at term suggesting a strong association between PPROM and placental abruption [61].

A few links may connect placental abruption with PPROM. An inflammation at the maternal–fetal interface is a likely common pathway leading to preterm birth in both placental abruption and PPROM cases [121,122,123]. It may be manifested both by the presence of a bacterial infection commonly seen in the setting of PPROM or a subclinical sterile inflammation present in chronic placental abruption [83]. Another link between placental abruption and PPROM is through THR with its numerous molecular effects described above. THR output from an acute or chronic placental abruption eventually promotes the breakdown of the fetal membranes.

### 3.5. The Role of Tissue Factor in Placental Abruption

Tissue factor is a transmembrane glycoprotein [124] and a primary initiator of hemostasis [77,125]. It is expressed by decidualized endometrial stromal cells, and is responsible for hemorrhage prevention throughout the pregnancy [124,126,127]. This phenomenon is the basis of the concept of obstetric hemostatic envelope [41]. TF is synthesized and expressed by a variety of normal and malignant cells, while the cyto- and syncytiotrophoblast are ready to express TF under the influence of many pathogenic stimuli [41].

According to Kuczynski et al. (2002) [41], the highest TF levels were found in the decidua [77,125,127] and the whole placenta [41], high levels in the myometrium [41] and medium levels in the amniotic fluid [128,129]. TF was absent in the adjacent interstitial or extravillous cytotrophoblasts (CTBs) [127] and nearly absent in the blood serum of both pregnant and non-pregnant women [41]. Some researchers reported the increased levels of TF in the placenta in the case of PE [130]. As regards other tissues, the highest concentration of TF occurs in three organs: the brain, lungs and kidneys [131].

When the myometrium and placenta are destroyed during the placental abruption, the substances from the damaged tissues, TF and tissue factor pathway inhibitors, among others, are believed to be able to be reinfused to the circulation and affect the whole system [41,132,133]. The placenta and the myometrium are potentially relevant sources of TF, and the THR-generating potential of this TF is reflected in the profound hypofibrinogenemia and disseminated intravascular coagulation accompanying severe placental abruption.

### 3.6. The Role of Progesterone in Placental Abruption

In human pregnancy, the placenta-derived high progesterone concentrations at the maternal–fetal interface are stable until the delivery of the fetal–placental unit [50]. Progesterone maintains uterine quiescence [134,135], relaxes myometrial cells [54], decreases the expression of COX2 [54], IL-1β [54] and PAR1 [54], and protects the fetal membranes [55,81]. The interruption of this communication occurring in placental abruption may be limited to the maternal–fetal interface and is not reflected in the blood [54]. Therefore, it was hypothesized that we rather deal with functional P4 withdrawal than the actual progesterone decrease in term labor or placental abruption [50,134,136,137,138,139,140]. The withdrawal may be manifested as the decline in total progesterone receptor levels, a change in relation to progesterone receptor isoforms or an inhibition of the PR-binding activity. As regards the first described mechanism, the decrease in progesterone receptors of both types and total decidual progesterone receptor levels in placental abruption or after THR treating was actually confirmed by Lockwood et al. (2012) and Nishimura et al. (2020) [50,54] The responsibility for this decrease was assigned to THR in the mechanism of ERK1/2 MAPK phosphorylation, with p38 MAPK or p65 NF-κB pathways probably remaining uninvolved [50]. The second mechanism—a change in relation to progesterone receptor isoforms—was explained by the fact that only PR-B was a full-length progesterone receptor, responsible for the relaxatory actions of progesterone, with PR-A being merely its truncated form inhibiting the transcriptional activity of PR-B [141]. The described situation occurs during delivery, when increasing the ratio of PR-A to the PR-B level is induced by PGE2 and PGF2-α [142,143,144]. The third described mechanism—the inhibition of the receptor binding activity—was confirmed by Lockwood et al. (2012) [50] In pathological situations, progesterone partially blocks THR-induced myometrial contractions [54], reduces the effect of fetal membrane weakening [55], and reduces THR-enhanced CSF-2 secretion in term decidual cell (TDC) cultures [55]. Changes in progesterone receptor levels together with the relaxing effects of progesterone offer a potential target for progesterone treatment. This issue was widely discussed above in the paragraphs concerning thrombin-induced myometrial contractions and thrombin-induced fetal membrane weakening.

We summarize the most important molecular aspects in the maternal–fetal interface in placental abruption in Supplementary Materials—Figure S2.

## 4. Limitations and Risk of Bias Assessment

Due to the variety of the included study types, the risk of bias in the individual studies was assessed using the above-described OHAT Tool adapted by the authors for the needs of this review (https://ntp.niehs.nih.gov/ntp/ohat/pubs/riskofbiastool_508.pdf, accessed on 11 April 2021). The evaluation results are summarized in Supplementary Table S3.

The present review combined data across studies in order to estimate the relevant effects and molecular changes occurring in placental abruption. The main limitation of this review, as with any overview, is that in the methods, examined tissues or cell cultures, the equipment used is different across studies and the measured outcomes are sometimes contradictory. Therefore, the collective analysis of studies based on different methodologies poses a major challenge in terms of interpretation. Another significant limitation is the low number of patients from whom the samples were obtained. This does not allow the exclusion of the influence of individual predispositions. Moreover, the quality of the studies varied, so the publication bias might account for some of the effect we observed. The small quantities of the examined samples was a considerable problem in the majority of studies. Smaller trials are, in general, of less value for the formation of generalized conclusions and may have led to an overestimation of observed effects. Another limitation is the relative weakness of human studies compared to animal models, where timing in pregnancy and sampling after the stimulus to parturition can be tightly defined. Conversely, there were some cell culture studies included in the review in which the timing and exposure were easier to control. However, we have to remember that in vitro study results are not always parallel to clinical results.

## 5. Conclusions and Perspectives

The disruption of the immunological processes in the maternal–fetal interface plays a crucial role in the pathophysiology of placental abruption. The features of chronic or augmented non-infectious inflammation and increased decidual cytotoxic activity were found to be present in placental abruption samples in the reviewed studies. Such processes result in the improper timing and order of the reproductive tract changes and the preterm separation of the placenta. Tissue factor, thrombin and cytokines seem the most crucial mediators of those processes. However, still more data are necessary to extract details concerning their influence.

Co-signaling and immunomodulatory molecules constitute a very interesting and uncharted direction. They are mainly investigated in the context of carcinogenesis, but they may also influence the process of the maternal tolerance to fetal antigens. The discovery of clinically useful biomarkers determining whether processes leading to inflammatory and coagulation network disruption has already started would be crucial for defining high risk patients. The establishment of the mechanisms responsible for immune tolerance changes may help find anchor points for the immunotherapy of various high-risk pregnancies. It might lead to the concept of individualized medicine, as it seems impossible to find one therapy for the whole complex condition. The first steps have already been taken, but a great part of the necessary research still lies ahead.

## Figures and Tables

**Table 1:** The inclusion and exclusion criteria.

	Inclusion Criteria	Exclusion Criteria
Study status	Completed, Published	Unfinished, Unpublished
Study type	- Original article	- Review - Book chapters - Case reports - Case series - Expert opinions - Conference papers
Language	English	Other than English
Type of examination	Molecular examination of placenta	Histopathological, genetic or blood only examination
Sample origin	Human	Animal
Type of conception	Natural	IVF

**Table 2:** Thrombin effects on MMP expression.

	**Decidua**	**Fetal Membranes**
MMP-1 ^1,2^	↑ ^2^	MS↑ ^1^
MMP-2 ^3^		↑ ^3^
MMP-3 ^2,4,5^	→ ^5^/↑ ^2^	MS↑ ^1^
MMP-7 ^5^	→	
MMP-9 ^3,5,6^	↑ ^5^	↑ ^6^/→ ^3^
TIMP-3 ^6^		↓ ^6^

↑—increased expression, ↓—decreased expression, →—expression unchanged, MMP-1 = matrix metalloproteinase-2 = interstitial collagenase; MMP-2 = matrix metalloproteinase-2 = 72 kDa type IV collagenase = gelatinase A; MMP-3 = matrix metalloproteinase-3 = stromelysin-1; MMP-7 = matrix metalloproteinase-7 = matrilysin; MMP-9 = GELB = matrix metalloproteinase-9 = 92 kDa type IV collagenase = 92 kDa gelatinase or gelatinase B; MS = maternal side; TIMP3 = metalloproteinase inhibitor 3. ^1^ = [52]; ^2^ = [42]; ^3^ = [51]; ^4^ = [54]; ^5^ = [55]; ^6^ = [49].

**Table 3:** The effects of THR, LPS and the augmentation of the signal.

	THR	LPS	THR + LPS
IL-1β	⦿	⦿	⦿
IL-6	↑	↑	↑↑
IL-8	↑	↑	↑↑
IL-10	⦿	⦿	↑↑
IL-17	↓	⦿	↑↑
IFNγ	⦿	↑	↑↑
IP-10	↑	↑	⦿
TNFα	⦿	⦿	↑↑
G-CSF	↑	↑	↑↑
GM-CSF	⦿	⦿	↑↑
MCP-1	↑	↑	↑↑
GRO-α	↑	↑	↑↑
VEGF	⦿	⦿	↑↑
RANTES	⦿	⦿	⦿
MIP-1β	⦿	⦿	⦿

↑ = increased expression; ↑↑ = augmentation of an increase; ⦿ = no effect; MCP1 = CCL2 = C-C motif chemokine ligand 2 = monocyte chemoattractant protein 1; MIP-1-β = CCL4 = C-C motif chemokine ligand 4 = macrophage inflammatory protein 1-β; RANTES = CCL5 = C-C motif chemokine ligand 5 = regulated on activation, normal T-cell expressed and secreted; GRO-α = CXCL1 = C-X-C motif chemokine ligand 1 = growth regulated oncogene-α; IP-10 = CXCL10 = IFNγ induced protein 10RO; G-CSF = granulocyte-colony stimulating factor; GM-CSF = granulocyte-macrophage colony-stimulating factor; IFNγ = interferon gamma; IL-6 = interleukin 6; IL-8 = interleukin 8; IL-10 = interleukin 10; IL-17 = interleukin 17; IL-1β = interleukin-1β; LPS = lipopolysaccharides; THR = thrombin; TNFα = tumor necrosis factor α; VEGF = vascular endothelial growth factor.

## Data Availability

The data supporting the reported results can be found in searched databases.

## Supplementary Materials

The following are available online at https://www.mdpi.com/article/10.3390/ijms22126612/s1.
