# Supplementary material for: Molecular Changes on Maternal–Fetal Interface in Placental Abruption—A Systematic Review
Source: Int J Mol Sci. 2021 Jun 21;22(12):6612. doi: 10.3390/ijms22126612 (PMC8235312; doi:10.3390/ijms22126612)
Supplement: Supplementary file 1 [file ijms-22-06612-s001.zip › Supplementary Material 1.pdf]

Supplementary Materials I

Table S1. The search methods for used databases.

Database	Number of retrieved studies	Search strategy
PubMed	562	((placenta* AND abrupt*) OR abruptio placentae[MeSH]) AND (immun* OR Allergy and Immunology [MeSH] OR decidua* OR Decidua[MeSH] OR endometri* OR endometrium[MeSH] OR myometr* OR Myometrium[MeSH] OR trophoblast* Trophoblasts[MeSH] OR HEEC* OR cytotoxic* OR Cytotoxicity, Immunologic[MeSH] OR immunohistochemist* OR immunohistochemistry[Mesh] OR receptor* OR Receptors, Immunologic[MeSH] OR (immunolog* AND factor*) OR Immunologic Factors[MeSH] OR antigen* OR Antigens, Surface[MeSH] OR (maternal* AND fetal* AND interfac*))
Scopus	146	TITLE-ABS-KEY((placenta* AND abrupt*) AND (immun* OR decidua* OR endometri* OR myometr* OR trophoblast* OR HEEC* OR cytotoxic* OR immunohistochemist* OR receptor* OR antigen* OR (maternal* AND fetal* AND interfac*))) AND NOT INDEX(medline)

Table S2. Data collection from the included studies.

Authors	Year	Type of study	Hypothesis	Material examined	Molecules investigated	Effects observed in PA samples
Nishimura et al.	2020	Research article	Thrombin induces myometrial contractions by direct activation of myosin and indirect increase in prostaglandin synthesis.	2 myometrium samples from PA + human myometrium cells cultures of nonpregnant women + human myometrium cells cultures of pregnant woman	THR, PAR1, MLC2, MLCK, ROCK, COX2, PGE2, PGF2α, IL-1β, PTGER1, PTGER3, PTGFR, OXTR, GJA1, PR total, PR-B	↑THR, ↑PAR1, MLC2⚡□, MLCK⚡□, ROCK⚡□, ↑COX2, ↑PGE2, ↑PGF2α, ↑IL-1β, PTGER1 ◉, ↓PTGER3, ↓PTGFR, OXTR◉, GJA1◉, PR-A◉, ↓PR total, ↓PR-B
Sinkey et al.	2020	Research article	Thrombin-induced decidual CSF-2 acting via trophoblast-expressed CSF2R promotes abruption-related preterm birth by weakening FM.	10 decidua basalis samples from term birth (5 precontraction CC, 5 post-contraction CC), 8 decidua basalis samples from PTB, 8 decidua basalis samples from PA	CSF-2, CSF2Rα, ERK1/2 MAPK, NF-ϰB, p38 MAPK, STAT5, STAT3, Akt, NF-kB p65, MMP3, MMP7, MMP9, IL-1β, IL-6, IL-8	Decidua: ↑CSF-2, ERK1/2 MAPK ⚡□, NF-ϰB ⚡□, p38 MAPK ◉<br>Trophoblast: ↑CSF2Rα, STAT5 ⚡□, p38 MAPK ☒, STAT3 ◉, ERK1/2 ◉, AKT ◉, NF-kB p65 ◉<br>FM: ↑IL-1β*, ↑MMP9*, MMP3◉, MMP7 ◉, IL-6 ◉, IL-8 ◉
Singh et al.	2019	Research article	Inflammation was not a feature of placental abruption. It seems to be an acute event.	Placental samples:<br>13 non-labor controls<br>13 CA-PTL<br>8 I-PTL<br>7 PA-PTL	p65, AP-1, CREB, PGHS-2, OT, OTR, CX-43, IL-1β, IL-6, TNFα, IL-4, IL-10, IL-8, CXCL1, CXCL2, CCL2, CCL5, NFκB, MAPK/AP-1, CREB	Choriodecidua: CREB⚡□, IL-1β ◉, ↑IL-6, ↑TNFα, IL-4◉, ↑IL-10, ↑CXCL2<br>Amnion:<br>↑PGHS-2, ↑IL-1β, ↑TNFα , IL-4◉, IL-10◉, ↑IL-8, ↑CXCL2, ↑OT<br>Myometrium:<br>IL-1β ◉, IL-4◉, IL-10◉<br>Placenta:<br>IL-1β ◉, IL-4◉, IL-10◉, ↑CXCL2, ↓CCL2

Authors	Year	Type of study	Hypothesis	Material examined	Molecules investigated	Effects observed in PA samples
Mhatre et al.	2016	Research article	Thrombin, via PAR1 activation, augments LPS-induced HEEC production of chemokines. Inflammatory response is generated by synergistically PA and bacterial infection.	HEECs culture	THR, PAR1, PAR4, TLR4, LPS, IL-8, IL-1β, IL-6, IL-10, IL-12, IL-17, G-CSF, GM-CSF, IFNγ, MCP1/CCL2, MIP-1α/CCL3, MIP-1β/CCL4, RANTES/CCL5, TNFα, VEGF, GRO-α, IP-10/CXCL10, SOCS1, SOCS3	<u>POSSIBLE EFFECT:</u><br>PAR1 ϟ□, PAR4 ◉, ◉TLR4 expression, ◉IL-1β, ↑IL-6, ↑IL-8, ↑/◉IL-10, ↑/◉/↓IL-17, ↑/◉IFNγ, ↑/◉TNFα, ↑G-CSF, ↑/◉GM-CSF, ↑GRO-α, ↑MCP-1, ↑/◉VEGF, ◉MIP-1β, ◉RANTES, ↑/◉IP-10, ↑SOCS1, ↓SOCS3
Kumar et al.	2014	Research article	GM-CSF is a critical common intermediate in the thrombin and TNF FM weakening pathways. Thrombin applied to only the maternal side causes FM weakening in the same manner as when both sides were exposed.	Model of FM, maternal and fetal side presented separately	THR, TNFα, GM-CSF, PAI-1, cytokine array, protease array	<u>POSSIBLE EFFECT:</u><br><u>Maternal side</u>:<br>↑GM-CSF, ↓PAI-1, ↑MMP1, ↑MMP3, ↑Kallikriens 3, ↑Kallikriens 6, ↑Kallikriens 7<br><u>Fetal side</u>:<br>GM-CSF◉, ↓PAI-1
Puthiyachirakkal et al.	2013	Research article	Thrombin weakens the amnion ECM directly (rather than through PARs and also in killed amnion cells) and also activates MMPs.	Culture of amnion cells	THR, PARs, MMP2, MMP9, PGE2	<u>POSSIBLE EFFECT:</u><br>MMP2 ϟ□, MMP9 ◉

Authors	Year	Type of study	Hypothesis	Material examined	Molecules investigated	Effects observed in PA samples
Lockwood et al.	2012	Research article	Abruption-associated preterm delivery is initiated by functional progesterone withdrawal. Thrombin down-regulates decidual PR expression via ERK1/2 phosphorylation.	7 placenta tissue samples from PA<br>CONTROL: 7 placenta tissue samples from idiopathic PTB<br>Decidual cell cultures	PR (PR-A, PR-B), GR, NF-ϰB, p38 MAPK, ERK1/2 MAPK	Decidual cells: ↓PR, ↓PR-A, ↓PR-B, GR◉, ↑pERK1/2, total ERK1/2◉<br>Interstitial trophoblast: PR - absent, GR◉, total ERK1/2◉, pERK1/2◉<br>POSSIBLE EFFECT:<br>Decidual cells: NF-ϰB◉, p38 MAPK◉
Avagliano et al.	2011	Research article	An imbalance of COX level is not related to placental abruption. An imbalance of COX level may be present in cases with MA and CA but it is not related to placental abruption.	16 placentas with abruption<br>CONTROL: 26 normal placentas, 5 MA cases without abruption, 5 CA cases without abruption	COX1, COX2	COX1 ◉, COX2 ◉
Kumar et al.	2011	Research article	Thrombin weakens AM directly whereas cytokines weaken AM indirectly by causing the release of soluble intermediates from the choriodecidua.	FM model of full thickness FM and amnion separetely	THR, IL-1β, TNFα, NFκB inhibitor, LA, MMP9, TIMP3, PARP	POSSIBLE EFFECT:<br>↑MMP9, ↓TIMP3, ↑PARP cleavage
Snegovskikh et al.	2009	Research article	IL-1β stimulates VEGF and neuropilin-1/-2 in term decidual stromal cells. It provides a mechanism by which IAI can increase the risk of PA.	Decidual stromal cells culture (from term placentae)<br>14 placental tissue samples with and without intra-amniotic infection/inflammation	VEGF, Flt1, KDR2, neurolipin-1, neurolipin-2,	POSSIBLE EFFECT:<br>↑VEGF, Flt1 ◉, KDR2 ◉, ↑neurolipin-1, ↑neurolipin-2,

Authors	Year	Type of study	Hypothesis	Material examined	Molecules investigated	Effects observed in PA samples
Galazka et al.	2008	Research article	MT seems to be responsible for the proper coexistence between decidual cells and activated immune cells.	Decidual samples:<br>Eutopic (31 CC, 32 PA)<br>Ectopic (19 CC)	MT	↑MT
Norwitz et al.	2007	Research article	Abruption-associated decidual proteolysis and preterm labor is mediated primarily by thrombin-enhanced matrix metalloproteinase expression rather than an indirect effect on the plasminogen activator/inhibitor system	23 placenta samples from ECC	PAI-1, uPA, tPA	POSSIBLE EFFECT:<br>Decidual cells: ↑PAI-1, uPA◉, tPA◉
Wicherek et al.	2007	Research article	Placental abruption seems to be associated with excessive accumulation and activity of CD3+ and CD56+ cells in decidua, which processes might, in turn, result from an insufficient RCAS1 decidual level.	Decidual samples: 25 PA, 11 RPT<br>CONTROL: 30 PTB	RCAS1, CD3, CD56, CD69, CD25	↓RCAS1, ↑CD56, ↑CD3, ↑CD69, CD25◉
Wicherek et al.	2006	Research article	RCAS1 inhibits maternal immune response during gestation and parturition and has a potential involvement in the mechanism of PA.	(117 placenta tissue samples)<br>9 PA<br>CONTROL: 8 RPT, 40 VD, 60 ECC	RCAS1	↓RCAS1

Authors	Year	Type of study	Hypothesis	Material examined	Molecules investigated	Effects observed in PA samples
Lockwood et al.	2005	Research article	Thrombin initiates the influx of neutrophils into the decidua via IL-8 expression. It explains how abruption-associated PPROM promotes decidual neutrophil infiltration	Placental samples:<br>13 PA<br>2 PA & PPROM<br>8 controls - ECC<br>Decidual cells cultures from ECCs	IL-8, CD15	↑CD15<br><u>POSSIBLE EFFECT:</u><br>↑IL-8
Uszyński et al.	2004	Research article	Measured uPA and uPAR are present in all gestational tissues, in some in relatively high concentrations. Plasmin-dependent proteolytic system participate in obstetric complications such as placental abruption.	35 set of samples form CC (placenta, myometrium, foetal membranes, amniotic fluid, blood serum)<br>CONTROL: 20 non-pregnant blood serum samples	uPA, uPAR	<u>POSSIBLE EFFECT:</u><br>↑uPA, ↑uPAR
Di Simone et al.	2003	Research article	Homocysteine induced trophoblast apoptosis and significantly reduced human chorionic gonadotropin secretion. Trophoblast cell death might represent a pathogenic mechanism by which homocysteine may cause pregnancy complications such as PA.	Trophoblast cells culture	Homocysteine, M30, cytochrom c	<u>POSSIBLE EFFECT:</u><br>↑M30, ↑cytochrom c

Authors	Year	Type of study	Hypothesis	Material examined	Molecules investigated	Effects observed in PA samples
Kuczyński et al.	2002	Research article	The levels of TF in placenta and myometrium, are increased in relation to TF in blood plasma. The placenta and myometrium are potential sources of TF.	48 strips of placenta and myometrium from 48CC at term + blood serum samples<br>CONTROL: 20 non-pregnant blood serum samples	TF, TFPI	POSSIBLE EFFECT:<br>↑TF
Rosen et al.	2001	Research article	Thrombin-enhanced MMP-1 expression provides an explanation for the strong association between placental abruption and preterm membrane rupture	Endometrial cells - from 7 hysterectomies (myomas)<br>FM and decidual cells - from 3 ECC	MMP-1	POSSIBLE EFFECT:<br>↑MMP-1
Nakatsuka et al.	1999	Research article	CA and a portion of placental abruption may share a common cascade of placental injury. Nitric oxide and its metabolites may play an important role in this cascade.	Blood samples: 30 from healthy pregnant women, 16 from patients with CA<br>Placentas: 10 CA, 6 PA, 10 healthy term, 8 preterm from placenta previa or maternal heart disease	NO, NO syntase (eNOS, iNOS), nitrotyrosine, ICE	Placenta:↑iNOS, eNOS◉, ↑nitrotyrosine
Wang et al.	1987	Research article	Cocaine users showed a significantly lower total number of placental β-adrenergic receptors, μ-opiate receptors and δ-opiate receptors.	Placental samples:<br>Cocaine user mothers<br>Controls	β-adrenergic receptors, μ-opiate receptors, δ-opiate receptors	POSSIBLE EFFECT:<br>↓β-adrenergic receptors, ↓μ-opiate receptors, ↓δ-opiate receptors
Altshuler et al.	1983	Research article	HPL is elevated in placenta from PA patients.	Placental samples:<br>1 PA<br>1 PIH<br>1 FGR	HPL	↑HPL

Abbreviations: □ activation; ☒inhibition; ◉no effect; ↑ increased expression; ↓ decreased expression; * concentration dependent; Akt = PKB = Protein kinase B = serine/threonine-specific protein kinase; AP-1 = activator protein 1; CA = chorioamnionitis; CCL2 = MCP1 = C-C motif chemokine ligand 2 = monocyte chemoattractant protein 1; CCL3 = MIP-1-α = C-C motif chemokine ligand 3 = macrophage

inflammatory protein 1-α; CCL4 = MIP-1-β = C-C motif chemokine ligand 4 = macrophage inflammatory protein 1-β; CCL5 = RANTES = C-C motif chemokine ligand 5 = regulated on activation, normal T-cell expressed and secreted; CD15+ = neutrophils; CD25+ = IL-2 receptor; CD3+ = lymphocytes T; CD56+ = NK cells; CD69+ = evidence of CD56 activity; COX = cyclooxygenases (COX1, COX2); CREB = cAMP response element-binding protein; CSF-2 = GM-CSF = granulocyte-macrophage colony-stimulating factor = colony - stimulating factor 2; CSF2R = granulocyte-macrophage colony-stimulating factor receptor = colony-stimulating factor 2 receptor; CXCL1 = GRO-α = C-X-C motif chemokine ligand 1 = growth regulated oncogene-α; CXCL10 = IP-10 = IFNγ induced protein 10RO; CXCL2 = MIP2-α = Gro-β = Gro-2 = C-X-C motif chemokine ligand 2 = macrophage inflammatory protein 2-α = growth-regulated protein β = gro oncogene-2; ERK 1/2 MAPK = mitogen-activated protein kinase: subfamily of extracellular signal-regulated kinases; Flt1 = fms-like tyrosine kinase-1; G-CSF = granulocyte colony stimulating factor; GJA1 = CX-43 = gap junction alpha-1 protein = connexin 43; GM-CSF = granulocyte macrophage colony stimulating factor; GR = glucocorticoid receptor; HEECs = human endometrial endothelial cells; HPL = human placental lactogen; ICE = interleukin - 1β - converting enzyme = caspase-1; IFNγ = interferon gamma; IL - 4 = interleukin 4; IL-10 = interleukin 10; IL-12 = interleukin 12; IL-17 = interleukin 17; IL-1β = interleukin - 1β; IL-6 = interleukin 6; IL-8 = CXCL8 = interleukin 8; KDR2 = VEGFR-2 = kinase insert domain receptor 2 = vascular endothelial growth factor receptor 2; LA = lipoic acid; LPS = lipopolysaccharides; M30 = marker of apoptosis; MA = muscularised basal plate arteries; MLC2 = regulatory light chain of myosin II; MLCK = MLC kinase = mysoin light chain kinase; MMP-1 = matrix metalloproteinase-2 = interstitial collagenase; MMP-2 = matrix metalloproteinase-2 = 72 kDa type IV collagenase = gelatinase A; MMP-3 = matrix metalloproteinase-3 = stromelysin-1; MMP-7 = matrix metalloproteinase-7 = matrilysin; MMP-9 = GELB = matrix metalloproteinase-9 = 92 kDa type IV collagenase = 92 kDa gelatinase or gelatinase B; MT = metallothionein; NF-$\varkappa$B = nuclear factor kappa-light-chain-enhancer of activated B cells (NF-$\varkappa$B p65 - one of NF-$\varkappa$B components); NO = nitric oxide; NOS = NO syntetiase (iNOS = inducible NOS, eNOS = endothelial NOS); OT = oxitocine; OXTR = OTR = oxitocine receptor; p38 MAPK = mitogen-activated protein kinase: subfamily of p38 mitogen-activated protein kinases ; p65 = transcription factor p65 = nuclear factor NF-kappa-B p65 subunit; PAI = plasminogen activator inhibitors (PAI-1, PAI-2); PAR1 = F2R = protease-activated receptor 1; PAR4 = protease-activated receptor 4; PARP = poly (ADP-ribose) polymerase; PARs = protease-activated receptors = thrombin receptors (PAR1, PAR2, PAR3, PAR4); PGE2 = prostaglandin E2; PGE2-R = PTGER1, PTGER3 = prostaglandin E2 receptors; PGF2α = prostaglandin F2α; PGF2α-R = PTGFR = prostaglandin F2α receptor; PR = PgR = progesterone receptor; PR-A = PgR-A = progesterone receptor A; PR-B = PgR-B = progesterone receptor B; PTB = preterm birth; PTGS2 = PGHS-2 = COX2 = prostaglandin-endoperoxidase synthase-2; PTL = preterm labour (CA-PTL = Chorioamnionitis PTL, I-PTL = idiopathic PTL, PA-PTL = PA PTL); RCAS1 = receptor-binding cancer antigen expressed on SiSo cells; ROCK = Rh—associated protein myosin light chain kinase; SOCS = suppressors of cytokine signaling (SOCS1, SOCS3); STAT3 = Signal transducer and activator of transcription 3; STAT5 = Signal transducer and activator of transcription 5; TDC = term decidual cells; TF = tissue factor; TFPI = tissue factor pathway inhibitor (TFP1, TFP2); THR = thrombin; TIMP3 = metalloproteinase inhibitor 3; TLR = Toll-like receptors; TNFα = tumor necrosis factor α; tPA = tissue plasminogen activator; uPA = urokinase plasminogen activator; uPAR = urokinase plasminogen activator receptor; VEGF = vascular endothelial growth factor

Table S3. Risk of bias assessment. *Note: This table is based on the Office of Health Assessment and Translation (OHAT) Risk of Bias Rating Tool for Human and Animal Studies [1].*

**Newcastle - Ottawa Quality Assessment Scale[1] - modified**	**Altshuler et al. 1983**	**Wang et al. 1987**	**Nakatsuka et al. 1999**	**Rosen et al. 2001**	**Kuczyński et al. 2002**	**DiSimone et al. 2003**	**Uszyński et al. 2004**	**Lockwood et al. 2005**	**Wicherek et al. 2006**	**Wicherek et al. 2007**	**Norwitz et al. 2007**	**Galazka et al. 2008**	**Snegovskikh et al. 2009**	**Kumar et al. 2011**	**Avagliano et al. 2011**	**Lockwood et al. 2012**	**Puthiyachirakkal et al. 2013**	**Kumar et al. 2014**	**Mhatre et al. 2016**	**Singh et al. 2019**	**Sinkey et al. 2020**	**Nishimura et al. 2020**
1. Was administered dose or exposure level adequately randomized?	N/A	N/A	N/A	N/A	N/A	N/A	N/A	N/A	N/A	N/A	N/A	N/A	N/A	N/A	N/A	N/A	N/A	N/A	N/A	N/A	N/A	N/A
2. Was allocation to study groups adequately concealed?	N/A	N/A	N/A	N/A	N/A	N/A	N/A	N/A	N/A	N/A	N/A	N/A	N/A	N/A	N/A	N/A	N/A	N/A	N/A	N/A	N/A	N/A
3. Did selection of study participants result in the appropriate comparison groups?	N/A	+	+	N/A	+	N/A	+	+	+	+	N/A	+	N/A	N/A	++	++	N/A	N/A	N/A	++	N/A	N/A
4. Did study design or analysis account for important confounding and modifying variables?	-	+	+	N/A	+	N/A	+	+	++	++	N/A	++	N/A	N/A	++	+	N/A	N/A	N/A	++	N/A	N/A
5. Were experimental conditions identical across study groups?	N/A	N/A	N/A	++	N/A	++	N/A	++	N/A	N/A	++	N/A	++	++	N/A	++	++	++	++	N/A	++	++

6. Were research personnel blinded to the study group during the study? (or in in-vitro studies were the measurements done by computer/robotic system?)	N/A	N/A	N/A	++	N/A	++	N/A	++	N/A	N/A	++	N/A	++	++	N/A	++	++	++	++	N/A	++	++
7. Were outcome data complete without attrition or exclusion from analysis?	N/A	NR/-	NR/-	+	+	+	+	+	+	+	+	+	+	+	NR/-	+	+	+	+	++	+	+
8. Can we be confident in the exposure characterization?	+	+	++	++	+	+	+	++	++	++	++	++	++	+	++	++	++	++	++	++	+	+
9. Can we be confident in the outcome assessment (including blinding of assessors)?	+	+	++	++	+	++	++	++	+	+	++	+	++	++	+	++	++	++	++	+	++	++
10. Were all measured outcomes reported?	+	+	+	+	+	+	+	+	++	++	++	++	++	+	+	++	++	++	++	++	++	++
11. Were there no other potential threats to internal validity	-	-	+	+	+	+	+	+	+	+	++	+	+	+	++	+	++	++	+	++	+	+

https://ntp.niehs.nih.gov/ntp/ohat/pubs/riskofbiastool_508.pdf *accessed on 11th April 2021.*
