# Supplementary material for: Molecular Changes on Maternal–Fetal Interface in Placental Abruption—A Systematic Review
Source: Int J Mol Sci. 2021 Jun 21;22(12):6612. doi: 10.3390/ijms22126612 (PMC8235312; doi:10.3390/ijms22126612)
Supplement: Supplementary file 1 [file ijms-22-06612-s001.zip › Supplementary Material 2.pdf]

Supplementary Materials II

Table S4. The main changes of inflammatory molecules on the maternal-fetal interface.

	Myometrium	Decidua	Placenta	Fetal membranes	HEECs
IL-1β[1,4,5,6]	⦿1 / ↑[*4]	⦿[^1]	⦿[1]	↑[#1,5]	⦿[*6]
IL-4[1]			⦿		
IL-6[1,5,6]		↑[^1]		⦿[5]	↑[*6]
IL-8[1,3,5,6]			↑[*3]	↑[#1] / ⦿[5]	↑[*6]
IL-10[1,6]	⦿	↑[^]	⦿	⦿[#1]	↑/⦿[*6]
IL-17[6]					↑/ ⦿/↓[*6]
CXCL1[6]					↑[*6]
CXCL2[1]		↑[^]	↑	↑[#1]	
CXCL10[6]					↑/⦿[*6]
CCL2[1,6]			↓[1]		↑[*6]
CCL4[6]					⦿[*6]
CCL5[6]					⦿[*6]
SOCS1[6]					↑[*6]
SOCS3[6]					↓[*6]
VEGF[6,7]		↑[*7]			↑/⦿[*6]
Neurolipin-17		↑[*7]			
Neurolipin-17		↑[*7]			
KDR2[7]		⦿[*7]			
Flt-1[7]		⦿[*7]			
RCAS1[2,8]		↓[2]	↓[8]		
MT[9]		↑[9]			
TNFα[1,6]		↑[^]			↑/⦿[*6]
IFNγ[6]					↑/⦿[*6]
CD3[2]		↑[2]			
CD15[3]		↑[3]	↑[3]		
CD25[2]		⦿[2]			
CD56[2]		↑[2]			
CD69[2]		↑[2]			

	Myometrium	Decidua	Placenta	Fetal membranes	HEECs
PGE2[4]	↑*				
PGF2α[4]	↑*				
PTGFR[4]	↓*				
PTGER3[4]	↓*				
PTGER1[4]	◉*				
PR[2]		↓			
PR-A[2]		↓			
PR-B[2]		↓			
CSF-2[5,6,10]		↑		MS↑*[10] / FS↑*[10]	↑/◉*[6]
G-CSF[6]					↑*[6]
ERK 1/2 MAPK[2,5]		ϟ□[2,5]			
p38 MAPK[2,5]		⊠ [5,*2]			
NF-κB[2,5]		ϟ□[5] / ⊠*[2]			

Abbreviations: □ activation; ⊠inhibition; ◉no effect; ↑ increased expression; ↓ decreased expression; * possible effect; ^ choriodecidua; # amnion; CCL2 = MCP1 = C-C motif chemokine ligand 2 = monocyte chemoattractant protein 1; CCL4 = MIP-1-β = C-C motif chemokine ligand 4 = macrophage inflammatory protein 1-β; CCL5 = RANTES = C-C motif chemokine ligand 5 = regulated on activation, normal T-cell expressed and secreted; CD15+ = neutrophils; CD25+ = IL-2 receptor; CD3+ = lymphocytes T; CD56+ = NK cells; CD69+ = evidence of CD56 activity; CSF-2 = GM-CSF = granulocyte-macrophage colony-stimulating factor = colony - stimulating factor 2; CXCL1 = GRO-α = C-X-C motif chemokine ligand 1 = growth regulated oncogene-α; CXCL10 = IP-10 = IFNγ induced protein 10RO; CXCL2 = MIP2-α = Gro-β = Gro-2 = C-X-C motif chemokine ligand 2 = macrophage inflammatory protein 2-α = growth-regulated protein β = gro oncogene-2; ERK 1/2 MAPK = mitogen-activated protein kinase: subfamily of extracellular signal-regulated kinases; Flt1 = fms-like tyrosine kinase-1; FS = fetal side; HEECs = human endometrial endothelial cells; IFNγ = interferon gamma; IL-4 = interleukin 4; IL-10 = interleukin 10; IL-17 = interleukin 17; IL-1β = interleukin - 1β; IL-6 = interleukin 6; IL-8 = CXCL8 = interleukin 8; KDR2 = VEGFR-2 = kinase insert domain receptor 2 = vascular endothelial growth factor receptor 2; MMP-1 = matrix metalloproteinase-2 = interstitial collagenase; MMP-2 = matrix metalloproteinase-2 = 72 kDa type IV collagenase = gelatinase A; MMP-3 = matrix metalloproteinase-3 = stromelysin-1; MMP-7 = matrix metalloproteinase-7 = matrilysin; MMP-9 = GELB = matrix metalloproteinase-9 = 92 kDa type IV collagenase = 92 kDa gelatinase or gelatinase B; MS = maternal side; MT = metallothionein; NF-κB = nuclear factor kappa-light-chain-enhancer of activated B cells (NF-κB p65 - one of NF-κB components); p38 MAPK = mitogen-activated protein kinase: subfamily of p38 mitogen-activated protein kinases ; PGE2 = prostaglandin E2; PGE2-R = PTGER1, PTGER3 = prostaglandin E2 receptors; PGF2α = prostaglandin F2α; PGF2α-R = PTGFR = prostaglandin F2α receptor; PR = PgR = progesterone receptor; PR-A = PgR-A = progesterone receptor A; PR-B = PgR-B = progesterone receptor B; RCAS1 = receptor-binding cancer antigen expressed on SiSo cells; SOCS = suppressors of cytokine signaling (SOCS1, SOCS3); TIMP3 = metalloproteinase inhibitor 3; TNFα = tumor necrosis factor α; VEGF = vascular endothelial growth factor

[1] = (1); [2] = (2); [3] = (3); [4] = (4); [5] = (5); [6] = (6); [7] = (7); [8] = (8); [9] = (9); [10] = (10); [11] = (11); [12] = (12); [13] = (13)

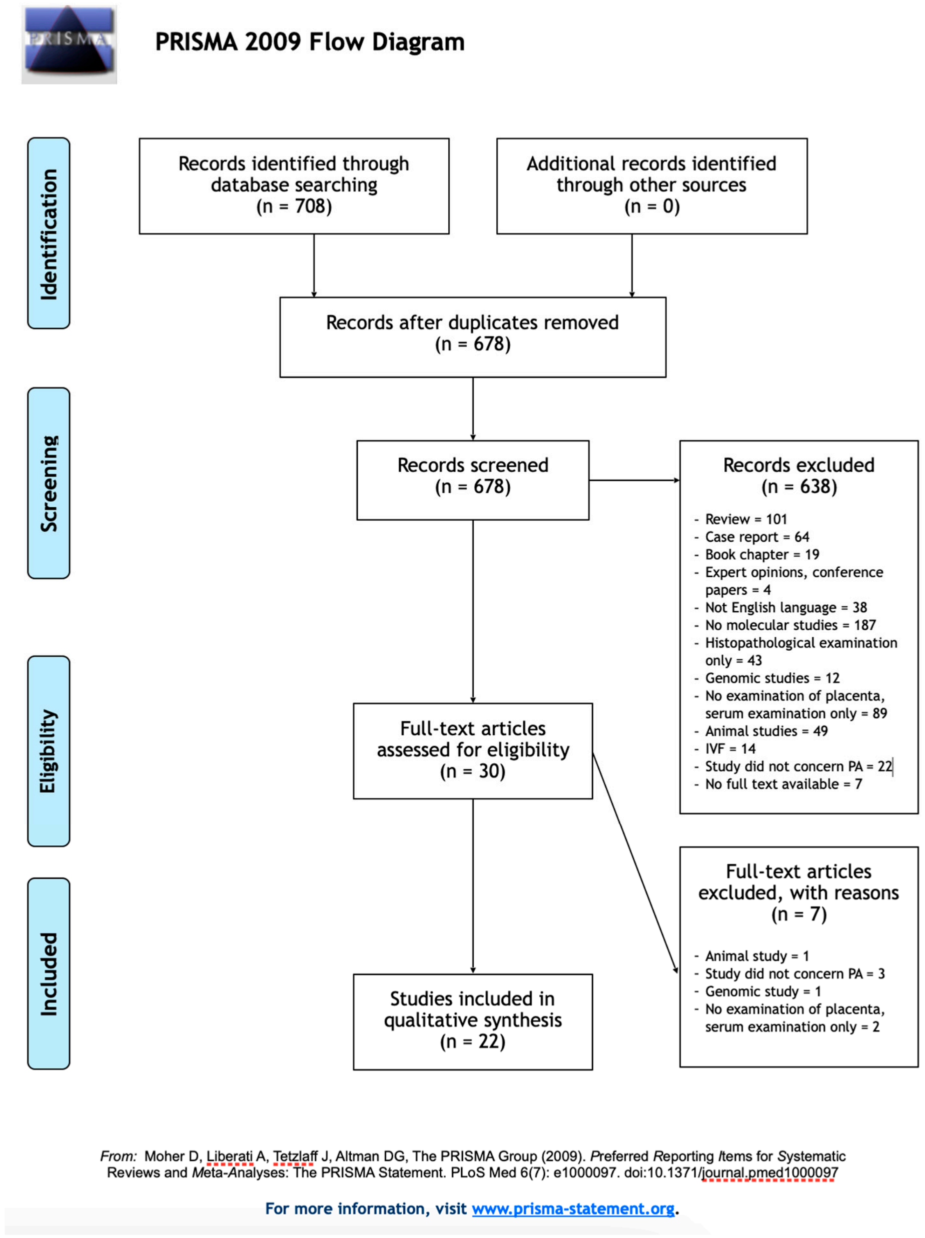

Figure S1. Prisma 2009 Flow Diagram.

References

1. Singh N, Herbert B, Sooranna G, Das A, Sooranna SR, Yellon SM, et al. Distinct preterm labor phenotypes have unique inflammatory signatures and contraction associated protein profilesdagger. Biol Reprod. 2019;101(5):1031-45.
2. Wicherek L, Galazka K, Lazar A. RCAS1 decidual immunoreactivity during placental abruption: immune cell presence and activity. Am J Reprod Immunol. 2007;58(1):46-55.
3. Lockwood CJ, Toti P, Arcuri F, Paidas M, Buchwalder L, Krikun G, et al. Mechanisms of abruption-induced premature rupture of the fetal membranes: thrombin-enhanced interleukin-8 expression in term decidua. Am J Pathol. 2005;167(5):1443-9.
4. Nishimura F, Mogami H, Moriuchi K, Chigusa Y, Mandai M, Kondoh E. Mechanisms of thrombin-Induced myometrial contractions: Potential targets of progesterone. PLoS One. 2020;15(5):e0231944.
5. Sinkey RG, Guzeloglu-Kayisli O, Arlier S, Guo X, Semerci N, Moore R, et al. Thrombin-Induced Decidual Colony-Stimulating Factor-2 Promotes Abruption-Related Preterm Birth by Weakening Fetal Membranes. Am J Pathol. 2020;190(2):388-99.
6. Mhatre MV, Potter JA, Lockwood CJ, Krikun G, Abrahams VM. Thrombin Augments LPS-Induced Human Endometrial Endothelial Cell Inflammation via PAR1 Activation. Am J Reprod Immunol. 2016;76(1):29-37.
7. Snegovskikh VV, Schatz F, Arcuri F, Toti P, Kayisli UA, Murk W, et al. Intra-amniotic infection upregulates decidual cell vascular endothelial growth factor (VEGF) and neuropilin-1 and -2 expression: implications for infection-related preterm birth. Reprod Sci. 2009;16(8):767-80.
8. Wicherek L, Klimek M, Dutsch-Wicherek M, Kolodziejski L, Skotniczny K. The molecular changes during placental detachment. Eur J Obstet Gynecol Reprod Biol. 2006;125(2):171-5.
9. Galazka K, Pitynski K, Skret-Magierlo J, Mach P, Knafel A, Sikora J, et al. The increase in metallothionein and ectopic decidual immunoreactivity with respect to the progression of labor at term and the lack of analogical changes in placental abruption. Am J Reprod Immunol. 2008;60(3):204-13.
10. Kumar D, Moore RM, Nash A, Springel E, Mercer BM, Philipson E, et al. Decidual GM-CSF is a critical common intermediate necessary for thrombin and TNF induced in-vitro fetal membrane weakening. Placenta. 2014;35(12):1049-56.
11. Rosen T, Schatz F, Kuczynski E, Lam H, Koo AB, Lockwood CJ. Thrombin-enhanced matrix metalloproteinase-1 expression: a mechanism linking placental abruption with premature rupture of the membranes. J Matern Fetal Neonatal Med. 2002;11(1):11-7.
12. Kumar D, Schatz F, Moore RM, Mercer BM, Rangaswamy N, Mansour JM, et al. The effects of thrombin and cytokines upon the biomechanics and remodeling of isolated amnion membrane, in vitro. Placenta. 2011;32(3):206-13.
13. Puthiyachirakkal M, Lemerand K, Kumar D, Moore R, Philipson E, Mercer BM, et al. Thrombin weakens the amnion extracellular matrix (ECM) directly rather than through protease activated receptors. Placenta. 2013;34(10):924-31.
