# Supplementary figures and images for: Molecular Changes on Maternal–Fetal Interface in Placental Abruption—A Systematic Review
Source: Int J Mol Sci. 2021 Jun 21;22(12):6612. doi: 10.3390/ijms22126612 (PMC8235312; doi:10.3390/ijms22126612)

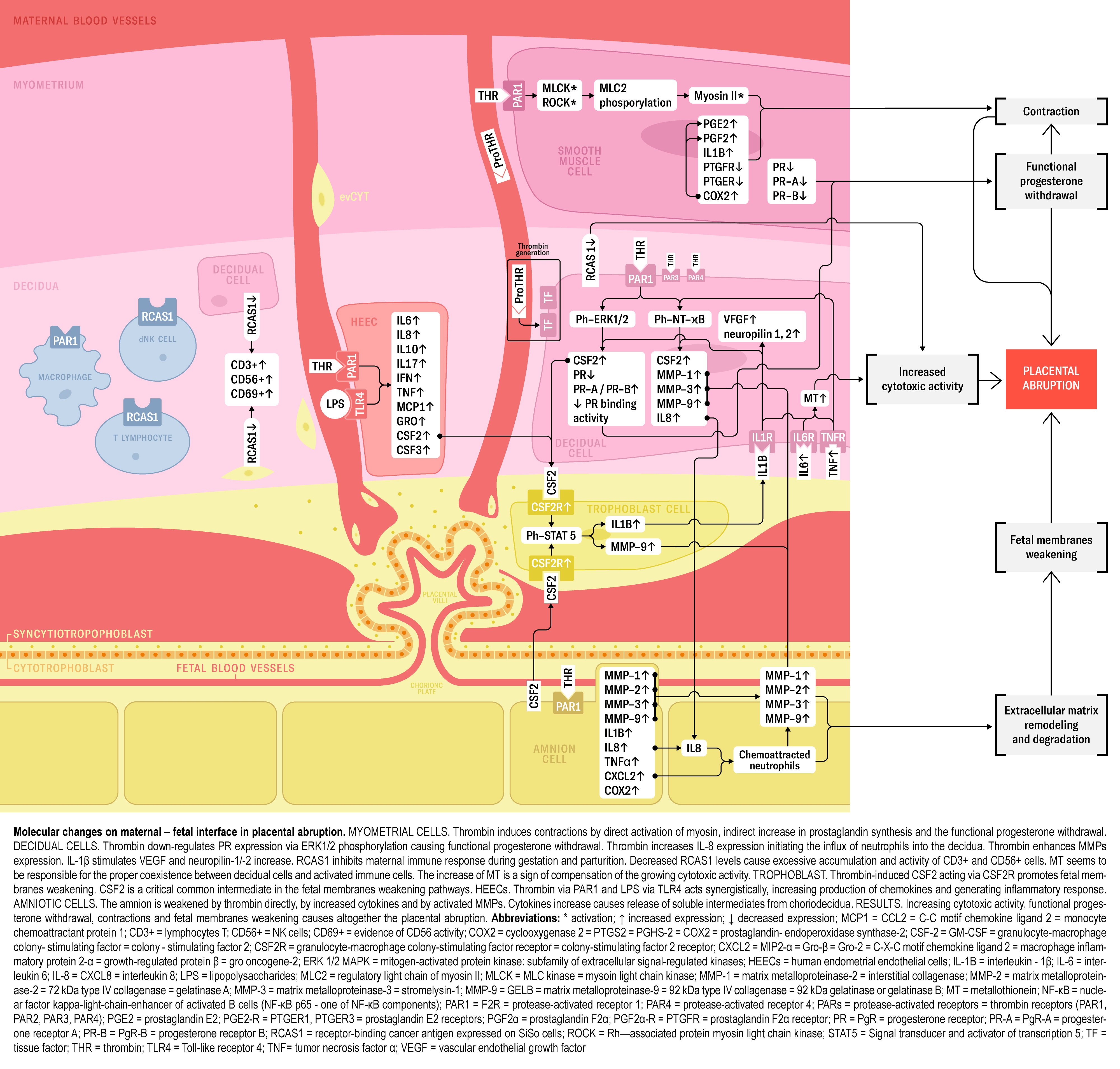

Supplement: Supplementary file 1 [file ijms-22-06612-s001.zip › Supplementary Material 3 (Figure S2).jpg]
